# MicroRNA dysregulation in pemphigus: a systematic review of expression profiles and molecular targets

**DOI:** 10.3389/fimmu.2026.1871963

**Published:** 2026-10-23

**Authors:** Safa Tahri, Ching Siang Tan, Liew Kai Bin, Ee-Ling Siew, Hatem Masmoudi, Olfa Abida, Johnathan Malagobadan, Nor Kamalia Binti Zahari

**Affiliations:** 1 Faculty of Medicine, University of Cyberjaya, Selangor, Malaysia; 2 School of Pharmacy, KPJ Healthcare University, Nilai, Malaysia; 3 Faculty of Pharmacy, University of Cyberjaya, Selangor, Malaysia; 4 ASASIpintar Unit, Pusat PERMATA@Pintar Negara, Universiti Kebangsaan Malaysia, Bangi, Malaysia; 5 Faculty of Health Sciences, Universiti Kebangsaan Malaysia, Kuala Lumpur, Malaysia; 6 Faculty of Medicine, University of Sfax, Sfax, Tunisia; 7 Research Laboratory LR18/SP12 “Autoimmunity, Cancer and Immunogenetics”, Habib Bourguiba Hospital, University of Sfax, Sfax, Tunisia; 8 School of Biomedical Engineering, University of Cyberjaya, Selangor, Malaysia

**Keywords:** microRNA, miR-146a-5p, miR-155-5p, miR-338-3p, miR-424-5p, pemphigus

## Abstract

Pemphigus is a severe autoimmune bullous disease characterized by the production of pathogenic autoantibodies leading to acantholysis. Emerging evidence suggests that microRNA regulate immune responses and may contribute to disease pathogenesis. However, an updated, integrative synthesis linking miRNA expression profiles to their molecular targets remains lacking. This review specifically addresses the expression profiles, target genes, and molecular pathways of miR-338-3p, miR-146a-5p, miR-155-5p and miR-424-5p, and their potential involvement in pemphigus. Original studies investigating microRNA expression or functional roles in patients with pemphigus or relevant experimental models were considered eligible. A literature search was conducted in Scopus, PubMed and ScienceDirect scientific databases between January 2000 and September 2025. Titles, abstracts, and full texts were screened, by two independent researchers according to predefined eligibility criteria. A total of nine studies were included in this review. Clinical evidence establishes miR-338-3p, miR-155-5p, and miR-424-5p as robust diagnostic biomarkers of active disease, with AUC values reaching 0.988. Notably, miR-338-3p and miR-155-5p levels show significant positive correlations with PDAI scores. Mechanistically, for miR-338-3p, the genes RNF114, TRADD, and RUNX1 have been experimentally validated as direct targets, linking this miRNA to Th1/Th2 imbalance and the suppression of FOXP3-mediated Treg activity. Similarly, miR-146a-5p has been validated to target the NF-κB adaptors TRAF6 and IRAK1, acting as an endogenous anti-inflammatory regulator. These findings identify specific miRNA axes as potential therapeutic targets and high-precision tools for clinical stratification and disease severity. However, further large-scale and mechanistic studies are needed to validate their functional target genes and clinical utility.

## 1 Introduction

Pemphigus is a bullous skin autoimmune disease (AID) characterized by the presence of intraepithelial blisters affecting both cutaneous and mucosal tissues (1). There are two main subtypes of Pemphigus: Pemphigus Vulgaris (PV) and Pemphigus Foliaceus (PF) (2). The core pathophysiology of Pemphigus involves the presence of circulating autoantibodies that target desmosomal adhesion molecules, particularly Desmogleins Dsg1 and Dsg3, which leads to a disruption of intercellular adhesion and progressive acantholysis (3). While the positive correlation between anti-Dsg titers and disease activity is statistically significant at the group level, individual-level discordance is frequently observed: patients may present with extensive active blistering in the context of relatively low autoantibody titers, or conversely maintain elevated titers during clinical remission (2, 4, 5). This dissociation underscores that autoantibody levels alone constitute an imprecise surrogate for disease activity and a poor predictor of relapse risk. Therapeutically, systemic corticosteroids remain the first-line mainstay, supplemented in moderate-to-severe disease by adjuvant immunosuppressants and, increasingly, by rituximab, a monoclonal anti-CD20 antibody that depletes autoreactive B cells (2, 6). Although rituximab has substantially improved remission rates and enabled corticosteroid tapering, a subset of patients experience relapse following initial B-cell reconstitution, and the long-term sequelae of repeated corticosteroid exposure, including osteoporosis, adrenal suppression, and metabolic complications, remain a substantial clinical burden (7). These limitations collectively reveal the insufficiency of a purely antibody-targeted therapeutic strategy and motivate the search for upstream regulatory mechanisms that govern autoreactive immune responses in pemphigus.

The pathological autoantibodies observed in pemphigus are not autonomous phenomena but rather mediated by autoreactive CD4^+^ T helper cells (8). A defining immunological feature of active PV is the imbalance between T helper 17 (Th17) cells and CD4^+^CD25^+^FOXP3^+^ regulatory T cells (Tregs). Studies demonstrate a marked expansion of Dsg3-responsive IL-17-secreting Th17 clones alongside a numerical and functional contraction of IL-10-producing Tregs in PV patients compared to healthy controls (9). Molecular analysis reveals elevated expression of the Th17-lineage transcription factor RORγt relative to the Treg master regulator FOXP3 in disease-active PBMCs populations, confirming a pro-inflammatory transcriptional bias (9). This Th17/Treg axis dysregulation sustains a pro-inflammatory cytokine milieu, enriched in IL-17, IL-6, IL-21, and TNF-α that amplifies autoreactive B-cell responses, impairs keratinocyte barrier function, and promotes lesional persistence (8, 9). In addition, broader adaptive and innate immune pathways, including Th1/Th2 polarization and Toll-like receptor–driven NF-κB signaling, are implicated in autoimmune inflammation and its regulation by microRNAs (3–5).

The recognition that pemphigus pathogenesis involves a systemic regulatory crisis at the T-cell level, rather than simple B-cell autoreactivity, fundamentally reframes the disease pathophysiology and points toward mechanisms that operate upstream of antibody production, namely, the post-transcriptional control of gene expression networks governing immune cell differentiation and function.

MicroRNAs (miRNAs) are a class of small, endogenously encoded, single-stranded non-coding RNA molecules of approximately 18–25 nucleotides that exert widespread regulatory control over gene expression at the post-transcriptional level (10). A defining feature of miRNA-mediated regulation is its combinatorial architecture: a single miRNA species may target hundreds of distinct mRNA transcripts, enabling coordinated regulation of entire gene-expression programs and signaling networks rather than isolated molecular targets (10). Amidst the diverse landscape of dysregulated microRNA profiles in autoimmune blistering diseases, increasing attention has focused on miR-338-3p, miR-146a-5p, miR-155-5p, and miR-424-5p due to their pivotal roles in immune regulation and epidermal integrity. In systemic lupus erythematosus (SLE) and rheumatoid arthritis (RA), aberrant expression of miRNAs including miR-155, miR-146a, miR-21, and miR-let-7 in T cells has been linked to defective DNA methylation, altered T-cell signaling, dysregulated cytokine production, and impaired regulatory T-cell function (11) (**Figure 1**).

**Figure 1:**
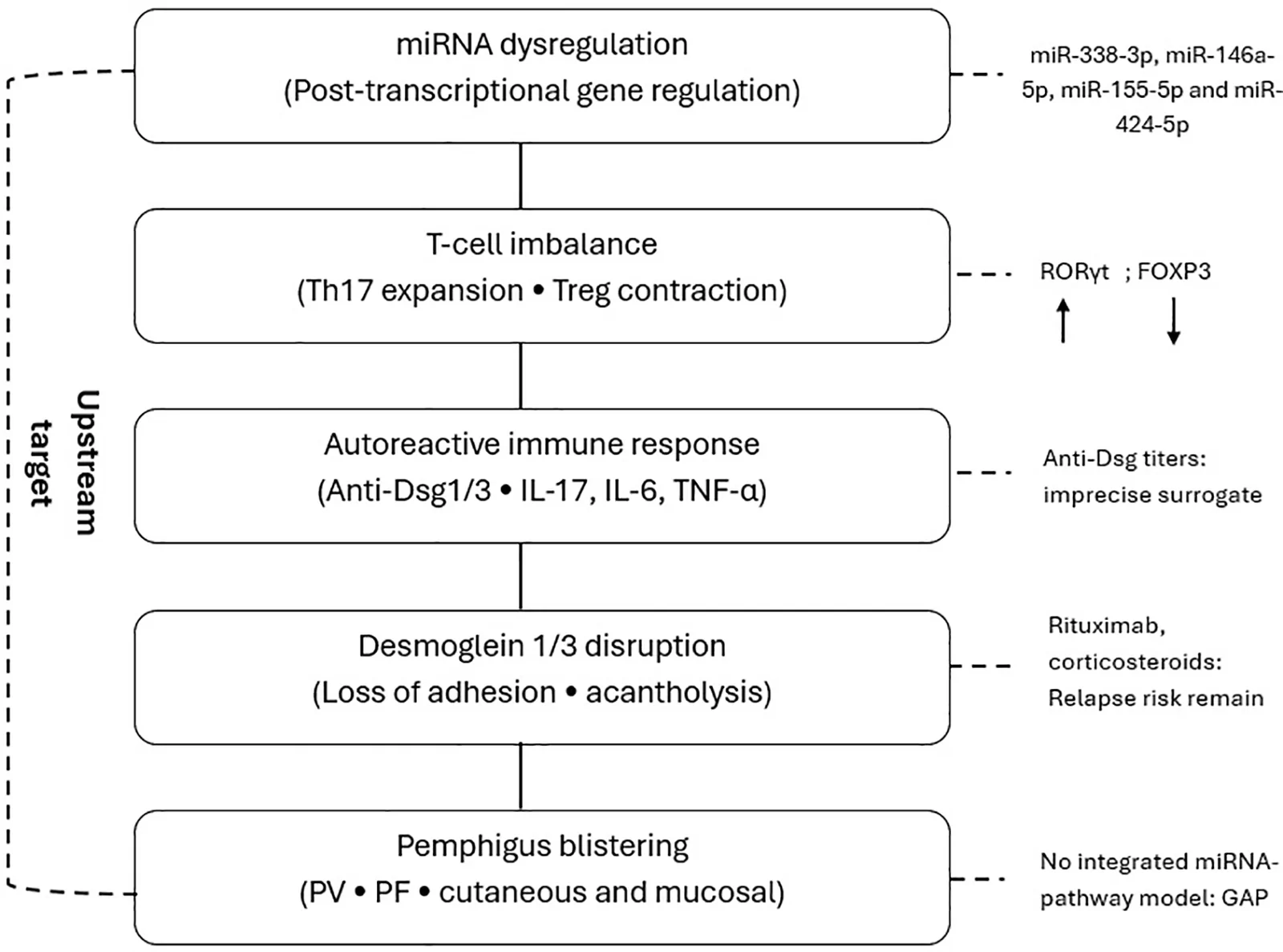
Pemphigus cascade outline.

In the context of pemphigus, an emerging body of evidence implicates specific miRNA species as functional modulators of the immunological and keratinocyte-intrinsic processes underlying disease pathogenesis. Studies in PF and PV have demonstrated significant dysregulation of miR-338-3p, miR-146a-5p, miR-155-5p and miR-424-5p, with expression profiles varying across disease states, analyzed biological compartments and treatment status (12–14). Despite the existence of an updated review by Ravazi et al. (12), which included eight studies and discussed the evidence for miRNA involvement in pemphigus based on expression patterns, the review did not integrate miRNA expression, regulatory targets, and signaling pathways interactions into a comprehensive model of pemphigus pathogenesis (12).

This systematic review provides a comprehensive synthesis of the available evidence regarding the role of four miRNAs: miR-338-3p, miR-146a-5p, miR-155-5p, and miR-424-5p, in pemphigus pathogenesis and clinical course. These four candidates were selected based on their identification across independent pemphigus studies and their established roles in immune regulatory pathways relevant to autoimmunity.

The specific objectives of this review are fourfold. First, to characterize the expression profiles of these miRNAs across biological compartments, disease subtypes, and disease phases (active versus remission). Second, to evaluate reported correlations between miRNA levels and established clinical and serological markers of disease activity, including the Pemphigus Disease Activity Index (PDAI), the Autoimmune Bullous Skin Disorder Intensity Score (ABSIS), and anti-Dsg1/anti-Dsg3 antibody titers. Third, to assess the diagnostic and prognostic utility of these miRNAs through available receiver operating characteristic (ROC) analyses and biomarker performance metrics. Fourth, to critically appraise the mechanistic evidence for each miRNA, explicitly distinguishing computationally predicted from experimentally validated target-pathway interactions. By integrating these lines of evidence, this review aims to identify the most promising directions for future mechanistic research and translational applications.

## 2 Methods

### 2.1 Eligibility criteria

Eligibility criteria were established to target microRNAs with the most robust functional and clinical evidence in pemphigus pathogenesis. Initially, a comprehensive literature search was conducted to identify all microRNAs (miRNAs) reported to be differentially expressed and associated with signaling pathways in the context of pemphigus. miR-146a-5p, miR-155-5p, and miR-424-5p were selected for in-depth analysis and studies were included based on the following criteria:

1. Research articles exploring miRNA in pemphigus.

2. Consistent dysregulation reported across at least two independent cohorts.

3. Investigation of miRNA expression in human subjects with pemphigus.

4. The existence of experimentally validated and/or predicted molecular targets and/or associated signaling pathways in the context of pemphigus.

5. Publication in English.

### 2.2 Exclusion criteria

studies unrelated to pemphigus or miRNAs, non-original articles (reviews, editorials, and letters), conference abstracts, animal or *in vitro* studies without human samples, insufficient data, and duplicate records. Preprints were considered if sufficient methodological information was available for quality assessment.

### 2.3 Information sources and search strategy

This systematic review was performed in accordance with the Preferred Reporting Items for Systematic Reviews and Meta-Analyses (PRISMA) 2020 guidelines (15). A systematic literature search was conducted across the PubMed, ScienceDirect and Scopus databases, published between January 2000 and September 2025. The search strategy was constructed using a combination of Medical Subject Headings (MeSH) and free-text keywords related to the condition (“Pemphigus,” “Pemphigus Vulgaris,” “Pemphigus Foliaceus,” “Autoimmune blistering diseases”) and the molecule of interest (“miRNA”). eg., (“miR-146a-5p,” “microRNA-146a,” “miRNA-146a”, “miRNA”). Boolean operators (AND, OR) were applied to refine the results. For example, one of the core search strings used in PubMed was: (“Pemphigus”[MeSH] OR “Pemphigus Vulgaris” OR “Pemphigus Foliaceus”) AND (“miR” OR “microRNA” OR “miRNA”).

### 2.4 Selection and data collection

Following the initial search, all retrieved records were imported into Zotero to identify and remove duplicates through automated deduplication tools. Subsequently, two reviewers independently screened the titles and abstracts of the remaining records against our predefined inclusion and exclusion criteria. Any disagreements regarding the eligibility of a study were resolved through discussion with a third independent reviewer to reach a final consensus. A total of nine studies were included. For each study, we documented the primary miRNA candidate (miR-338-3p, miR-146a-5p, miR-155-5p, or miR-424-5p) and categorized the findings based on the following parameters:

- Study characteristics including reference (Author/Year), pemphigus subtype (PV or PF), and population details (cohort size and demographics),

- Biological context including sample type (e.g., serum, PBMCs, or skin) and the associated disease phase (active or remission).

- Quantitative data on miRNA expression, including reported levels (mean ± SD) and fold changes between groups.

- Clinical relevance including diagnostic performance metrics (AUC, sensitivity, specificity) and correlations with established clinical indices (PDAI and ABSIS), along with longitudinal associations with treatment response when available.

- Finally, mechanistic insights were documented by identifying reported target genes and pathways, while distinguishing between experimentally validated interactions (such as dual-luciferase assays or RT-qPCR) and those based solely on in silico predictions (e.g., TargetScan or miRanda).

### 2.5 Quality assessment

The methodological quality of all included studies was evaluated independently using the Newcastle-Ottawa Scale (NOS) for case-control studies.

A quantitative meta-analysis was not performed because the included studies were not sufficiently homogeneous in design to permit pooled effect-size estimation. Sources of heterogeneity include (1): biological compartment (serum, whole PBMCs, isolated CD4^+^ T cells, lesional skin, plasma exosomes) (2); pemphigus subtype and disease phase composition (3); outcome metrics (fold-change from microarray, RT-qPCR relative expression, AUC/sensitivity/specificity, Spearman correlation coefficients with different clinical indices (PDAI, ABSIS, PAAS); and (4) inclusion of one preprint (16) alongside peer-reviewed studies.

## 3 Results

### 3.1 Characteristics of the studies

Initially, 99 articles were identified after searching three electronic databases: Scopus (n=67), PubMed (n=19), and ScienceDirect (n=13). Following the screening process outlined in the PRISMA flow diagram (**Figure 2**), nine articles were selected for inclusion in this systematic review. One of the included studies was a preprint that provided sufficient methodological detail to allow eligibility assessment (16). All included studies had a case-control design, published between 2017 and 2025. No additional eligible studies were identified through manual searching. The characteristics of each study regarding sample type, pemphigus type, and study population are summarized in **Table 1**. All included studies were assessed for methodological quality using the Newcastle-Ottawa Scale (NOS) for case-control studies. The mean NOS total score of the retained studies was 7.33 (range 7–9), indicating satisfactory methodological quality across all included studies (**Table 2**). Among the nine studies, three investigated PV exclusively, three examined both PV and PF across active disease and remission groups, one focused solely on PF, one did not specify pemphigus subtype, and one examined mucosal pemphigus vulgaris (MPV). The biological samples analyzed varied across studies and included PBMCs, CD4^+^ T cells isolated from PBMCs, serum, and lesional skin biopsies. Study populations ranged from small exploratory groups to larger validation cohorts, with sample sizes varying from 10 to 86 participants. Several studies stratified participants into active disease, remission, and healthy control groups, and some additionally included patients with other bullous dermatoses such as bullous pemphigoid, lichen planus, dermatitis herpetiformis, and cicatricial pemphigoid as comparative groups.

**Figure 2:**
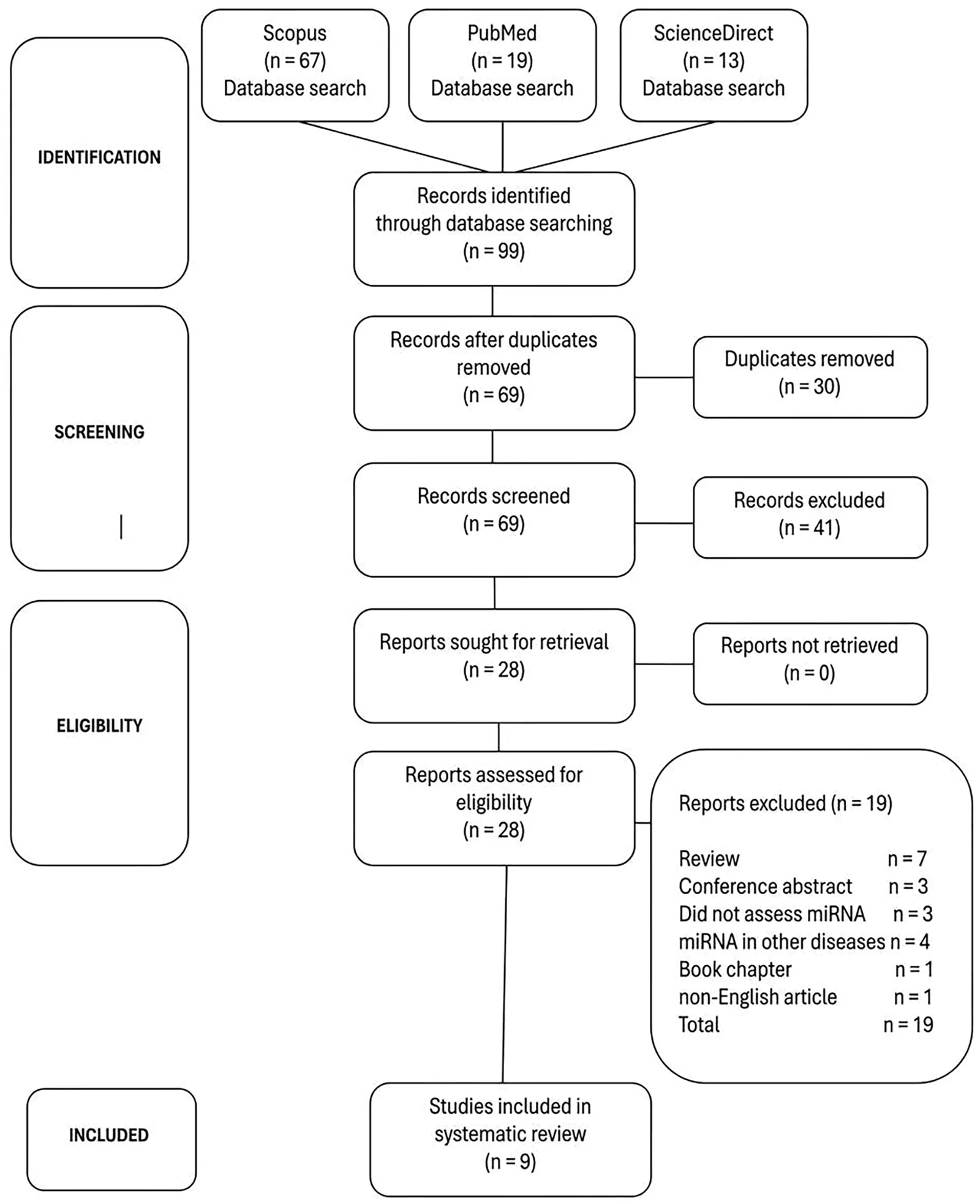
PRISMA 2020 flow diagram of selection of studies for the systematic review.

**Table 1:** Characteristics of the included studies.

Reference	Sample type	Pemphigus type	Study population
M. Wang et al., 2017 (17)	PBMCs	Pemphigus (not specified)	**Microarray analysis:** • 3 patients with active pemphigus • 3 Healthy controls **Validation with RT-qPCR:** • 9 patients with pemphigus • 9 Healthy controls
Lin et al., 2018 (18)	PBMCs	PV/PF	**35 pemphigus patients:** • 32 PV + 3 PF • 30 active (new blisters, no prior immunosuppressant) • 5 remission (no new blisters > 6 months) • 7 bullous pemphigoid (BP) • 33 Healthy controls
Liu et al., 2018 (19)	PBMCs	PV	• 11 PV patients • 11 Healthy controls
Xu et al., 2020 (20)	CD4^+^ T cells isolated from PBMCs	PV	• 26 PV patients • 20 Healthy controls
He et al., 2022 (21)	Serum	PV	• 22 PV patients • 15 mucous membrane pemphigoid (MMP) patients • 10 Healthy controls
Khabou et al., 2023 (22)	PBMCs and lesional skin biopsy	PF	**20 PF patients/7 Healthy controls:** • Group 1: 12 untreated, newly diagnosed (follow-up at 6 weeks [n=3] and/or 3 months [n=5]) • Group 2: 5 treated patients with mild pemphigus • Group 3: 3 patients with moderate-to-severe pemphigus
Valentino et al., 2023 (25)	Serum	Mucosal PV (MPV)	• 5 MPV patients • 5 Healthy controls
Teplyuk et al., 2024 (23)	PBMCs	PV/PF	• Active disease group (n=10) • Remission group (n=3) • Healthy controls (n=9)
Teplyuk et al., 2025 (24)	PBMCs	PV/PF	**G1 (n=37):** Active pemphigus (28 PV, 9 PF; 19 relapse, 18 new onset) **G2 (n=20):** Remission > 1 year (14 PV, 6 PF) **G3 (n=20):** Healthy controls **G4 (n=9):** Other bullous dermatoses (4 BP, 2 lichen planus, 2 dermatitis herpetiformis, 1 cicatricial pemphigoid)

PBMCs, peripheral blood mononuclear cells; PV, pemphigus vulgaris; PF, pemphigus foliaceus; MPV, mucosal pemphigus vulgaris; MMP, mucous membrane pemphigoid; BP, bullous pemphigoid; HC, healthy controls; RT-qPCR, reverse transcription quantitative polymerase chain reaction; G, group; n, number of subjects included in each group.

**Table 2:** Newcastle-Ottawa scale (NOS) quality assessment of eligible studies.

Study	Selection (max 4★)	Comparability (max 2★)	Exposure (max 3★)	Total (max 9★)
Teplyuk et al., 2025 (16)	**★★★★**	**★**☆	**★★★**	**8/9**
Teplyuk et al., 2024 (23)	**★★★★**	**★**☆	**★★**☆	**7/9**
Valentino et al., 2024 (25)	**★★★**☆	**★★**	**★★**☆	**7/9**
Khabou et al., 2023 (22)	**★★★★**	**★★**	**★★★**	**9/9**
He et al., 2022 (21)	**★★★**☆	**★**☆	**★★★**	**7/9**
Xu et al., 2020 (20)	**★★★**☆	**★**☆	**★★★**	**7/9**
Liu et al., 2018 (19)	**★★★**☆	**★**☆	**★★★**	**7/9**
Lin et al., 2018 (18)	**★★★**☆	**★★**	**★★★**	**7/9**
Wang et al., 2017 (17)	**★★★**☆	**★**☆	**★★★**	**7/9**

The Newcastle-Ottawa Scale (NOS) was applied to assess the methodological quality of nine studies included in this systematic review. NOS evaluates three domains: Selection (max 4 stars), Comparability (max 2 stars), and Outcome/Exposure (max 3 stars), yielding a maximum total of 9 stars per study. Quality thresholds: Low risk of bias ≥7★ | Moderate risk 4–6★ | High risk ≤3★ |.

#### 3.1.1 miR-338-3p in pemphigus: expression profiles, clinical correlations, and molecular mechanisms

##### 3.1.1.1 Expression profiles across biological compartments and disease subtypes

miR-338-3p was investigated across eight studies included in this review (**Tables 3**, **4**). The first evidence of miR-338-3p dysregulation in pemphigus originated from a miRNA microarray study conducted by Wang et al. (17). Analysis of PBMCs from three patients with active pemphigus and three HC identified 124 differentially expressed miRNAs, of which miR-338-3p was the second most upregulated, with a fold change of approximately 1,452 (*p* = 0.0021) (17). This result has been confirmed across multiple independent cohorts. In PBMCs, Lin et al. (18) reported significantly elevated miR-338-3p in 30 patients with active pemphigus compared to 33 HC (*p* < 0.001) (18). Liu et al. (19) similarly demonstrated significant upregulation in PBMCs from 11 PV patients versus 11 HC (p < 0.01). Xu et al. (20), working with isolated CD4^+^ T cells, confirmed elevated miR-338-3p in 26 PV patients compared to 20 HC (p < 0.0001), representing the only study in this review to characterize expression at the level of a specific T-cell subset. In the serum compartment, He et al. (21) detected significant elevation of miR-338-3p among 12 measured miRNAs in 22 MPV patients relative to 10 HC (P < 0.001).

**Table 3:** Summary of studies reporting miR-338-3p expression, clinical correlations, diagnostic performance, and clinical significance in pemphigus.

Reference	Expression levels (Mean ± SD)	Disease association	Correlation with disease activity	Correlation with treatment response	Diagnostic performance	Clinical significance
Wang et al., 2017 (17)	NR	Upregulated in active pemphigus vs. HC^a^ Fold change: 1,451.8 (P = 0.0021)	N/A	N/A	N/A	Discovery study; identified miR-338-3p as a top candidate for subsequent investigation
Lin et al., 2018 (18)	Active: 5.55 ± 3.64 Remission: 1.80 ± 1.18 BP: 0.31 ± 0.18 HC: 1.31 ± 0.96	Upregulated in active disease vs. all comparator groups (P < 0.001)	Positive correlation with PDAI and ABSIS	Significant progressive decrease during effective therapy (P < 0.001) Significant decline at 2 weeks (earlier than anti-Dsg3) Continued decline at 6 weeks	AUC: 0.8919 Cutoff: 2.676 Sensitivity: 86.67% Specificity: 87.88% (Active pemphigus vs. HC)	Proposed as a diagnostic biomarker and early treatment-response marker in pemphigus; responds to therapy earlier than conventional serology
Liu et al., 2018 (19)	NR	Upregulated in active PV vs. HC (P < 0.01)	Positive correlation with PAAS^b^ (r = 0.700, P = 0.016); and anti-Dsg3 titers (r = 0.619, P = 0.042)	N/A: study design excluded patients on recent treatment	Diagnostic performance parameters not reported	Proposed as therapeutic target in PV
Xu et al., 2020 (20)	NR	Upregulated in active PV vs. HC (P < 0.0001)	Positive correlation with PDAI (r = 0.755, P < 0.001); and anti-Dsg3 titers (r = 0.546, P = 0.004)	Significant decrease in effective treatment group after 2-week GCS therapy (P < 0.01)	NR	Potentially useful for early therapeutic monitoring
He et al., 2022 (21)	NR	Upregulated in PV vs. NC (P < 0.001)	N/A	N/A	Not analyzed for miR-338-3p specifically	Potential diagnostic utility
Khabou et al., 2023 (22)	Blood miR-338: G1: 0.003 ± 0.0008 G2: 0.0025 ± 0.0004 G3: 0.0058 ± 0.003 Skin miR-338: G1: 0.085 ± 0.018 G2: 0.059 ± 0.031 G3: 0.31 ± 0.14	No significant baseline difference between PF patients (G1) and HC in blood or skin	No significant correlation with PDAI for blood miR-338 (r = 0.25, P = 0.29)	Significant decrease in blood miR-338 at 3 months of effective therapy (1.47 ± 0.56 vs. 0.12 ± 0.039; P = 0.036) Not significant at 6 weeks (P = 0.27) No significant change in skin levels at either time point	N/A	Only PF-specific study; results do not support miR-338-3p as a diagnostic or severity marker in endemic PF;
Teplyuk et al., 2024 (23)	Active: 8.64 ± 5.72 Remission: 3.38 ± 1.44 HC: 1.48 ± 1.12	Upregulated in active disease vs. HC (P = 0.002)	Strong positive correlation with PDAI: r = 0.834, P < 0.001	N/A	Diagnostic performance not reported	confirmed PDAI correlation;
Teplyuk et al., 2025 (24)	active: 9.53 ± 6.24 remission: 2.13 ± 1.47 HC: 1.42 ± 1.06 other bullous: 2.10 ± 1.85	Upregulated in active disease vs. all other groups (P < 0.0001)	Strong positive correlation with PDAI: r = 0.828, P < 0.0001	Significant 1.36-fold decrease after 3-week inpatient GCS therapy (P = 0.0001)	AUC: 0.9711 Cutoff: > 3.753 Sensitivity: 94.59% Specificity: 92.00%	a diagnostic marker, disease severity indicator, and treatment-response monitor in PV/PF

AUC, area under the curve; BP, bullous pemphigoid; GCS, glucocorticosteroids; HC, healthy controls; NR, not reported; N/A, not assessed; PAAS, pemphigus area and activity score; PDAI, pemphigus disease area index; PF, pemphigus foliaceus; PV, pemphigus vulgaris.

^a^ Microarray fold change reported.

**Table 4:** Experimentally validated and computationally predicted molecular targets and signaling pathways for miR-338-3p, miR-146a-5p, miR-155-5p, and miR-424-5p in pemphigus.

miRNA	Reference	Experimentally validated targets/pathways	Computationally predicted targets/pathways	Validation and prediction methods
**miR-338-3p**	Lin et al., 2018 (18)	**Target gene: RNF114** Ring Finger Protein 114	*No computationally predicted targets reported separately; RNF114 emerged from*	**Prediction (candidate selection):** miRwalk and TargetScan databases used to identify candidate target genes. **Experimental validation:** **(1**) miR-338-3p mimic/inhibitor transfection into primary PBMCs (2) RT-qPCR (mRNA level) (3) Western blot (protein level) (4) Dual-luciferase reporter assay with wild-type and mutant RNF114 3′UTR constructs
		(RNF114) confirmed as a direct target of miR-338-3p. Overexpression of miR-338-3p significantly suppressed RNF114 at both mRNA and protein levels *in vitro*.	*bioinformatics screening prior to experimental validation.*	
	Liu et al., 2018 (19)	**Target gene: TRADD** TNFR1-associated death domain protein (TRADD) confirmed as a direct target of miR-338-3p. Overexpression of miR-338-3p significantly reduced TRADD mRNA and protein *in vitro*; TRADD also downregulated in PV patient PBMCs *in vivo*.	*TRADD identified via bioinformatics screening prior to experimental validation;*	**Prediction (candidate selection):** MiRanda, PITA, and TargetScan databases (consensus approach). **Experimental validation:** **(1**) miR-338-3p mimic/inhibitor transfection into primary PBMCs (2) RT-qPCR (mRNA level) (3) Western blot (protein level) (4) Dual-luciferase reporter assay with wild-type and mutant TRADD 3′UTR constructs (5) ELISA for IFN-γ, IL-4, IL-10 cytokines in PBMC supernatant (6) CCK-8 cell viability assay
	Xu et al., 2020 (20)	**Target gene: RUNX1 → FOXP3 axis** Runt-related transcription factor 1 (RUNX1) confirmed as a direct target of miR-338-3p in CD4^+^ T cells. miR-338-3p overexpression suppressed RUNX1, which in turn reduced FOXP3 expression and decreased the frequency of FOXP3^+^ Treg cells. Negative correlation between miR-338-3p and RUNX1 confirmed in PV patients (r = −0.512, P < 0.001); positive correlation between RUNX1 and FOXP3 (r = 0.557, P < 0.001).	*RUNX1 identified as a putative target via TargetScan prior to experimental validation.* *Public mRNA microarray data from PV CD4^+^ T cells used to identify downregulated Treg-related genes (STAT5B, IL2RB, CCR9, RORA, RUNX1, IL17RB) as an independent supporting analysis.*	**Prediction (candidate selection):** TargetScan database; supported by public microarray gene expression analysis of CD4^+^ T cells. **Experimental validation:** **(1**) miR-338-3p mimic/inhibitor transfection into CD4^+^ T cells (isolated by magnetic beads, purity >95%) (2) RT-qPCR (mRNA level — RUNX1, FOXP3, RORC, IFNG) (3) Western blot (protein level — RUNX1, FOXP3) (4) Flow cytometry (FOXP3^+^ cell frequency) (5) Dual-luciferase reporter assay with wild-type and mutant RUNX1 3′UTR constructs
	Khabou et al., 2023 (22)	*No experimentally validated targets reported for miR-338-3p in this study.*	**Panther pathways (p < 0.05 only; no FDR correction applied):** • Cell cycle/PI3 kinase pathway • Insulin/IGF pathway – MAPK kinase/MAP kinase cascade	**Prediction method:** Diana-TarBase v7.0 and miRabel (both use experimentally validated interaction data, not pure sequence-based prediction). Venny used to identify consensus target genes across databases. Panther pathway enrichment analysis with Gene Ontology resource.
**miR-146a-5p**	Khabou et al., 2023 (22)	*No experimentally validated targets reported for miR-146a-5p in pemphigus patients in this study.*	**Panther pathways (p < 0.05 and FDR < 0.05; single significant pathway):** • Inflammation mediated by chemokine and cytokine signaling pathway **Additional pathways (p < 0.05; FDR not met):** • Interferon-gamma signaling pathway • p38 MAPK pathway • Toll receptor signaling pathway **Key predicted target genes (from literature-supported databases):** • CARD10 — mediator of NF-κB activation • IRAK1 — mediator of NF-κB activation • CCL5 — chemokine promoting immune cell infiltration in inflamed tissue	**Prediction method:** Diana-TarBase v7.0 and miRabel (experimentally validated interaction databases). Venny for consensus gene identification. Panther pathway enrichment Cytoscape interactome analysis (miR-146a/miR-155 antagonistic network).
**miR-155-5p**	Khabou et al., 2023 (22)	*No experimentally validated targets reported for miR-155-5p in pemphigus patients in this study. MyD88 is described as a validated miR-155 target based on published literature, not newly validated here.*	**Panther pathways (p < 0.05 and FDR < 0.05; shared with miR-17 and miR-21, >380 common genes):** • Cell cycle/PI3 kinase pathway • Cellular homeostasis via P53 signaling pathway and p53 pathway feedback loops 2 • Interferon-gamma signaling pathway • Inflammation mediated by chemokine and cytokine signaling pathway **Key targets discussed in interactome analysis:** • MyD88	**Prediction method:** Diana-TarBase v7.0 and miRabel. Venny for consensus gene identification. Panther pathway enrichment (p < 0.05 and FDR < 0.05). Cytoscape interactome analysis.
	He et al., 2022 (21)	*No experimentally validated targets reported. Bioinformatic analysis only.*	**KEGG pathway enrichment:** • T cell receptor signaling pathway • p53 signaling pathway • Cell adhesion molecules pathway • p38 MAPK signaling pathway **Shared hub genes (all six co-upregulated miRNAs; not specific to miR-155-5p):** • NOTCH1, SOCS3, BCL2	**Prediction method:** Four databases: miRDB, miRTarBase, miRWalk, and TargetScan. Intersection of database results used as candidate target genes; Cytoscape 3.7.1 for miRNA–target network construction. GO and KEGG enrichment analysis. Metascape for target gene network mapping.
**miR-424-5p**	Wang et al., 2017 (17)	*No experimentally validated targets reported.*	**Target gene prediction:** 3,539 predicted target genes identified across databases; 2,430 confirmed as expressed in humans. **GO biological process enrichment (P < 0.05, Benjamini < 0.05):** • Regulation of phosphorus/phosphate metabolic process • Regulation of kinase/protein kinase activity • MAPK cascade (44 genes) **KEGG pathway enrichment (P < 0.05, Benjamini < 0.05):** • T cell receptor signaling pathway (29 genes, P = 3.4E-4) • MAPK signaling pathway (52 genes, P = 4.8E-3) **Key candidate regulatory targets discussed:** • MAPKAPK3, MAPK/HSP27 CDC25B	**Prediction method:** miRanda, miRWalk, and TargetScan (intersection used for candidate genes). GO analysis (Gene Ontology resource). KEGG pathway analysis.

CCKR, cholecystokinin receptor; FDR, false discovery rate; GO, Gene Ontology; HIF, hypoxia-inducible factor; IGF, insulin-like growth factor; KEGG, Kyoto Encyclopedia of Genes and Genomes; MAPK, mitogen-activated protein kinase; NF-κB, nuclear factor kappa B; PBMCs, peripheral blood mononuclear cell; PF, pemphigus foliaceus; PI3K, phosphatidylinositol 3-kinase; PV, pemphigus vulgaris; RT-qPCR, reverse transcription–quantitative polymerase chain reaction; TRAF6, TNF receptor-associated factor 6; UTR, untranslated region.

^*^ The Panther pathway enrichment analysis in Khabou et al. (22) used Diana-TarBase v7.0 and miRabel, which catalogue experimentally validated miRNA–target interactions from the published literature. These databases do not perform *de novo* sequence-based target prediction; however, the pathway enrichment itself was computational and was not independently validated in pemphigus patient samples. Entries in the ‘Computationally predicted’ column for Khabou et al. (22) therefore reflect bioinformatic enrichment of published interaction data rather than experimental validation in pemphigus.

^†^ TRAF6 is a well-established validated target of miR-146a in multiple published studies across inflammatory conditions (including SLE and sepsis). In Khabou et al. (22), TRAF6 is discussed in the context of miR-146a/miR-155 interactome analysis based on literature evidence, not newly validated in pemphigus foliaceus patients.

^‡^ See Results (3.3) for the shared-gene basis of these pathway attributions.

^§^ NOTCH1, SOCS3, and BCL2 are hub genes identified by He et al. (21) across the network of all six miRNAs studied (miR-125b-5p, miR-155-5p, miR-181a/b-5p, miR-326, miR-584-5p). They are not specific predicted targets of miR-155-5p alone.

Two studies from the Russian group (16, 23) confirmed PBMCs upregulation in larger cohorts. Teplyuk et al. (23) reported significantly elevated miR-338-3p in a pilot cohort of 10 active patients versus nine HC (*p* = 0.002) (23), and Teplyuk et al. (16) extended these findings to 37 active patients, 20 in remission, 20 HC, and 10 patients with other bullous dermatoses (P < 0.0001) (16).

Regarding disease subtype, most of the evidence pertains to PV. The sole study addressing PF is that of Khabou et al. (22), who examined miR-338-3p in both PBMCs and lesional skin biopsies from 20 Tunisian endemic PF patients. In PBMCs, miR-338-3p did not reach statistical significance when comparing untreated *de novo* patients to HC (*p* = 0.55), however, the authors noted a significantly higher miRNA expression in PBMCs compared to skin biopsy samples (p = 0.028).

##### 3.1.1.2 Correlations with disease activity indices and serological markers

Lin et al. (18) demonstrated that miR-338-3p expression levels were significantly correlated with PDAI scores (*p* = 0.005) and with ABSIS scores (*p* < 0.001) (18).

Teplyuk et al. (23) also reported a strong positive correlation between miR-338-3p expression and PDAI in their pilot cohort (r = 0.834; *p* < 0.001) (23), and Teplyuk et al. (16) confirmed this in the larger prospective cohort (r = 0.828; *p* < 0.0001) (16). Xu et al. (20), working with CD4^+^ T-cell–derived miR-338-3p, reported a significant positive correlation with PDAI (r = 0.755; p < 0.001) and with anti-Dsg3 antibody titers (r = 0.546; p = 0.004), but not with anti-Dsg1 titers. Liu et al. (19) similarly observed positive correlations between miR-338-3p and PAAS scores (r = 0.700; *p* = 0.016) and anti-Dsg3 titers (r = 0.619; *p* = 0.042) *(*19).

##### 3.1.1.3 Diagnostic accuracy

Lin et al. (18) reported an AUC of 0.8919 for miR-338-3p in PBMCs distinguishing active pemphigus patients from HC, with 86.67% sensitivity and 87.88% specificity (18). Teplyuk et al. (16) reported a higher AUC of 0.9711 with 94.59% sensitivity and 92.00% specificity (16).

##### 3.1.1.4 Treatment response and longitudinal monitoring

Lin et al. (18) reported a statistically significant decrease in miR-338-3p expression after two weeks of effective treatment initiation (*p* < 0.001) (23 patients followed) and continued to decline significantly at six weeks (*p* < 0.001) (14 patients followed) (18). Importantly, anti-Dsg3 antibody levels did not decrease significantly at either time point, and anti-Dsg1 antibody levels reached significance only at the six-week time point (two weeks p=0.377; six weeks p=0.018) (18).

Xu et al. (20) further distinguished between treatment responders and non-responders in a cohort of 11 PV patients followed for two weeks of glucocorticoid therapy. Expression of miR-338-3p and PDAI scores decreased significantly in the effective treatment group (p < 0.01 and p<0.0001, respectively), whereas no apparent change was observed in the ineffective group.

Teplyuk et al. (16) reported a significant 1.36-fold decrease in miR-338-3p expression (*p* = 0.0001), by 26.7% between first and third weeks of systemic glucocorticoid therapy (rs = 0.8697, p < 0.0001) (16). Khabou et al. (22) observed a significant decrease in blood miR-338-3p levels at three months post-treatment (*p* = 0.036), but not at six weeks.

##### 3.1.1.5 Experimentally validated molecular targets and downstream pathways

Three studies identified molecular targets of miR-338-3p in pemphigus through *in vitro* functional validation. Lin et al. (18) identified Ring Finger Protein 114 (RNF114) as a direct target of miR-338-3p. Following computational screening using miRwalk and TargetScan, this gene was subjected to *in vitro* validation in PBMCs from HC. Transfection of miR-338-3p mimics produced a significant decrease in RNF114 mRNA (*p* < 0.0001) and protein levels (*p* < 0.01). This direct interaction was confirmed by dual-luciferase reporter gene assay: miR-338-3p mimics significantly suppressed luciferase activity driven by the wild-type RNF114 3′UTR, while mutation of the predicted binding site fully abolished this suppression (18).

Liu et al. (2018) identified TNFR1-associated death domain protein (TRADD), which regulates cell proliferation and apoptosis, as a second direct target, using a consensus bioinformatics approach across the MiRanda, PITA, and TargetScan databases. Direct binding was confirmed by dual-luciferase reporter assay, with complete abolition of the inhibitory effect upon mutation of the TRADD 3′UTR binding site (19). To investigate the effect of miR-338-3p on Th1/Th2 immune balance, the expression of miR-338-3p was detected by qRT-PCR after transfection with miR-338-3p mimics, miR-338-3p inhibitor or negative control oligonucleotides. *In vitro* overexpression of miR-338-3p in healthy PBMCs significantly decreased IFN-γ, a hallmark Th1 cytokine, and increased the Th2-associated cytokines IL-4 and IL-10, while inhibition of miR-338-3p in patient-derived PBMCs reversed this cytokine pattern (19).

Xu et al. (20) identified Runt-related transcription factor 1 (RUNX1) as a direct target of miR-338-3p in CD4^+^ T cells. A significant negative correlation between miR-338-3p and RUNX1 expression was observed in CD4^+^ T cells from PV patients (r = −0.512; *p* < 0.001), and the direct interaction was confirmed by dual-luciferase reporter assay demonstrating that miR-338-3p mimics suppressed wild-type but not mutant RUNX1 3′UTR-driven luciferase activity. Functional transfection experiments showed that miR-338-3p overexpression reduced both RUNX1 and FOXP3 mRNA and protein levels, while miR-338-3p inhibition restored their expression and increased the frequency of FOXP3^+^ T cells. FOXP3 and RUNX1 were both significantly decreased in PV CD4^+^ T cells relative to controls (*p* < 0.01), and RUNX1 expression correlated positively with FOXP3 (r = 0.557; *p* < 0.001).

##### 3.1.1.6 Computationally predicted targets and pathway enrichment

In contrast to the three studies above, Khabou et al. (22) relied exclusively on in silico methods to characterize the miR-338-3p regulatory network in PF. Using experimentally validated target databases (Diana-TarBase v7.0 and miRabel), the study identified common consensus targets subjected to Panther pathway enrichment analysis (FDR and P < 0.05). Identified pathways included cell cycle regulation, PI3 kinase signaling, and Wnt signaling, with additional associations involving angiogenesis and hypoxia-response pathways (HIF activation) (**Figures 3**, **4**).

**Figure 3:**
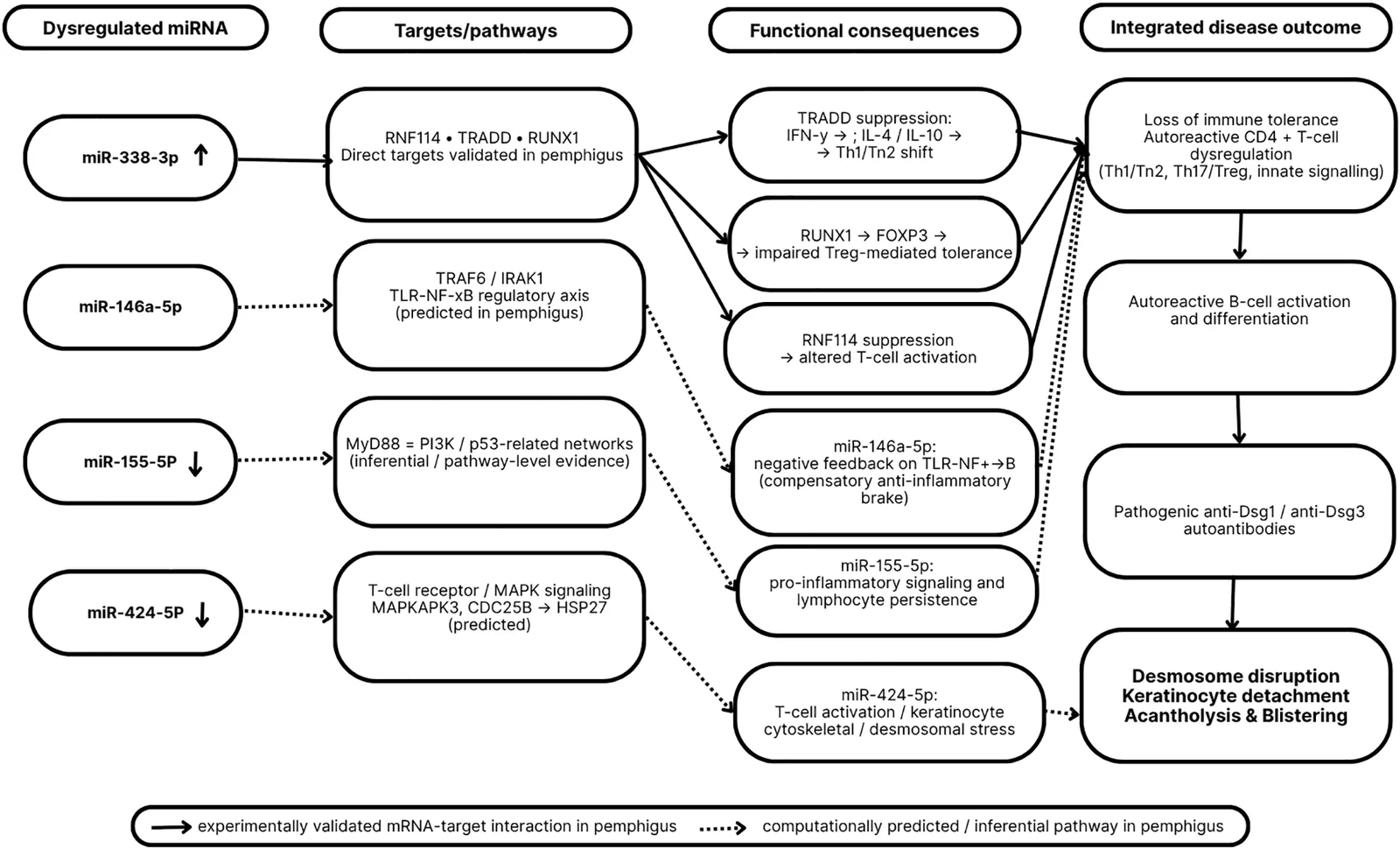
Integrated mechanistic model of miRNA-mediated Immunopathogenesis in pemphigus.

**Figure 4:**
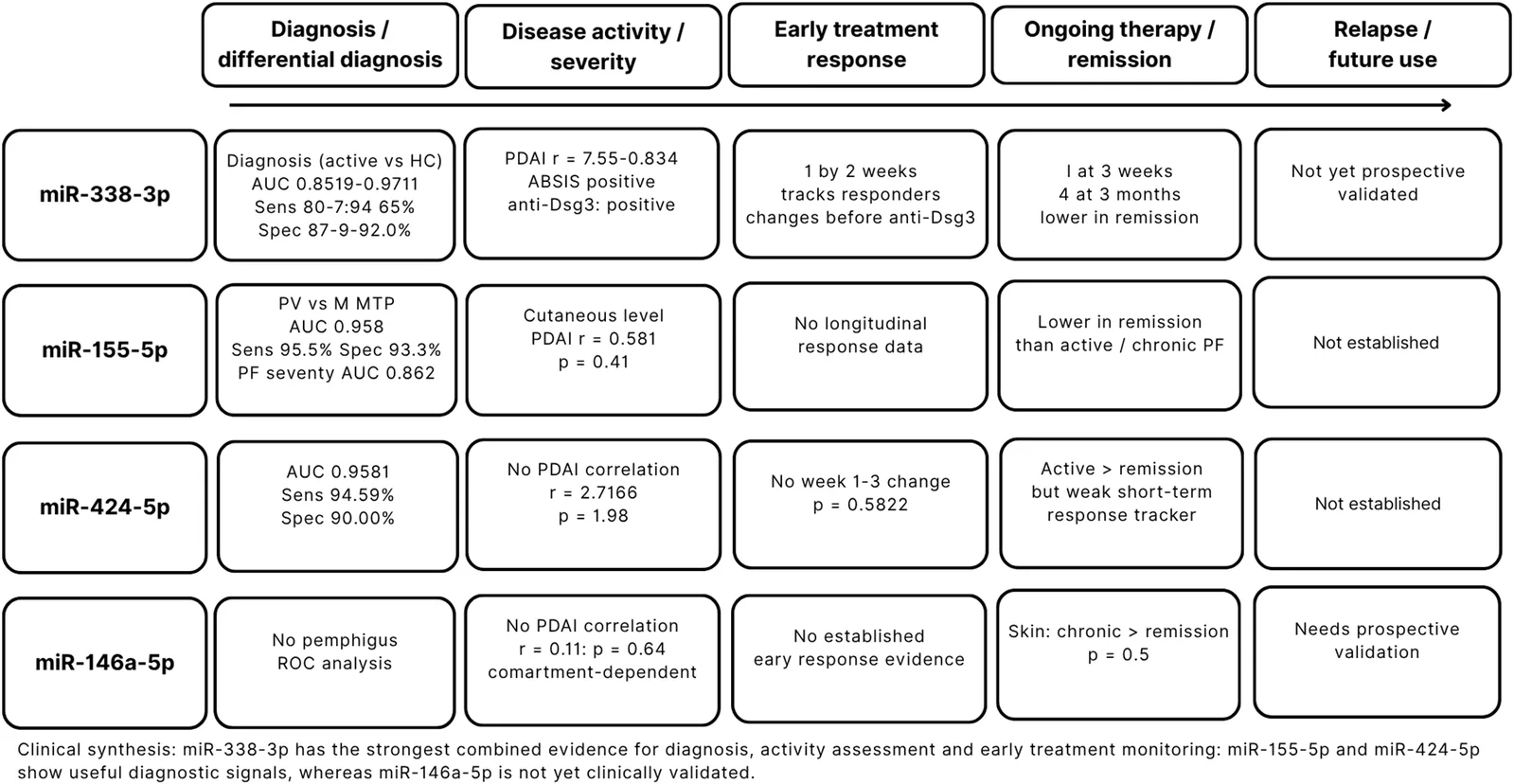
Clinical utility of miRNAs throughout the course of pemphigus.

#### 3.1.2 miR-146a-5p in pemphigus: expression profiles and mechanistic evidence

##### 3.1.2.1 Expression profiles of miR-146a-5p

Two studies included in this review reported on miR-146a-5p expression in pemphigus, each examining a distinct disease subtype and biological compartments.

Within the PF newly diagnosed untreated patients (G1), a statistically significant difference in miR-146a-5p expression was observed between biological compartments: levels were significantly lower in lesional skin biopsies than in PBMCs (p = 0.028). Blood miR-146a-5p levels did not differ significantly between untreated G1 patients and HC, despite a higher mean in patients (63.23 ± 22.48 vs. 15.65 ± 3.57; p = 0.2) (22).

In the lesional skin, miR-146a-5p expression differed significantly across PF clinical phases. Cutaneous miR-146a-5p levels were significantly higher in treated chronic patients compared to treated remittent patients (p = 0.05). No significant correlation was reported between blood/cutaneous miR-146a-5p expression levels and clinical and serological markers in PF (PDAI, anti-Dsg1 or anti-Dsg3 antibody titers) (cutaneous miR-146a-5p vs. PDAI: r = 0.11, p = 0.64) (22).

Additionally, cutaneous miR-146a-5p expression positively correlated with cutaneous miR-17, miR-21 and miR-155 levels across the lesional skin samples of G1, G2 and G3 patients, indicating a coordinated pattern of dysregulation among these miRNAs at the tissue level (22). In the HC group, a significant positive correlation was observed between miR-146a-5p and miR-155-5p (r = 0.821, p = 0.02) (22).

The only result reported by He et al. (21) was the significant miR-146a-5p upregulation in the serum of PV patients relative to HC (p < 0.001) (21).

##### 3.1.2.2 Mechanistic evidence: predicted and experimentally validated targets of miR-146a-5p

Mechanistic evidence for miR-146a-5p in the context of pemphigus was derived exclusively from Khabou et al. (22), who performed in silico target gene prediction and pathway enrichment analysis (methodology described previously).

The most significantly enriched pathway was the Toll receptor signaling pathway. Additional pathways meeting the p<0.05 threshold included the JAK/STAT signaling pathway, the IFNγ signaling pathway, and the inflammation mediated by chemokine and cytokine signaling pathway. At the level of individual target genes, Khabou et al. (22) identified several candidates within the enriched pathways including TRAF6, IRAK1 and CARD10, as well as PTGS2, which was mapped across three pathways (Toll receptor signaling, CCKR signaling map, and inflammation mediated by chemokine and cytokine signaling)(**Table 4**).

Beyond individual target–pathway mapping, Khabou et al. (22) performed a Cytoscape-based interactome analysis restricted to experimentally validated genes to examine the antagonistic relationship between miR-146a-5p and miR-155-5p in inflammation. This analysis identified TRAF6 as the central hub of the miR-146a-5p sub-network, interacting with several miR-155-5p targets, most notably MyD88 (22) (**Figures 3**, **4**).

#### 3.1.3 Expression profiles, biomarker assessment and mechanistic evidence of miR-155-5p in pemphigus

##### 3.1.3.1 Expression profiles of miR-155-5p

miR-155-5p expression and its related target genes were investigated in two studies included in this systematic review (21, 22).

In Khabou et al. (22), miR-155-5p expression was significantly lower in lesional skin biopsies than in PBMCs across the studied cohort (p = 0.028), consistent with the compartment pattern observed for the other studied miRNAs. Blood miR-155-5p did not differ significantly between untreated G1 patients and HC, despite a numerically higher mean in patients (19.4 ± 14.02 vs. 4.48 ± 2; p = 0.55) (22).

In the Khabou et al. cohort (22), cutaneous miR-155-5p was significantly higher in newly diagnosed untreated patients relative to treated patients in remission (p = 0.04). Among treated patients, those with chronic moderate-to-severe disease maintained significantly higher cutaneous levels than remittent patients (p = 0.02). Furthermore, a significant positive correlation between cutaneous miR-155-5p expression and the PDAI score was reported (r = 0.581, p = 0.04) (22).

In the He et al. (21) cohort, serum miR-155-5p was significantly upregulated in PV patients relative to HC (p < 0.001), and relative to MMP (p < 0.001) (21).

##### 3.1.3.2 Diagnostic and prognostic utility of miR-155-5p: ROC analyses

ROC curve analysis in Khabou et al. (22) showed that cutaneous miR-155-5p can discriminate between mild and moderate-to-severe chronic PF with an AUC of 0.862 (p = 0.01) with a cutoff of 0.04, and 100% sensitivity and 62.5% specificity.

In He et al. Study (21), serum miR-155-5p achieved strong individual diagnostic performance for discriminating PV from MMP (AUC = 0.985, cutoff 39.025) with 95.5% of sensitivity and 93.3% of specificity. In the serial combination model pairing miR-155-5p with miR-584-5p, the AUC was 0.977, with sensitivity of 95.5% and specificity of 100% (21).

##### 3.1.3.3 Mechanistic evidence: target genes and pathway associations of miR-155-5p

Panther pathway enrichment analysis for miR-155-5p, performed by Khabou et al. (22) identified five significantly enriched pathways: the Gonadotropin-releasing hormone receptor pathway, the PI3 kinase pathway, the p53 pathway feedback loops 2, the Hedgehog signaling pathway, and the 5-Hydroxytryptamine degradation pathway. These pathways were derived from consensus target genes shared across miR-17-5p, miR-21-5p, and miR-155-5p (>380 common genes), and cannot be attributed to miR-155-5p in isolation (22).

He et al. (21) predicted 1,030 candidate target genes for miR-155-5p; however, all subsequent GO and KEGG enrichment analyses were performed on the combined target pool of six miRNAs (miR-125b-5p, miR-155-5p, miR-181a-5p, miR-181b-5p, miR-326, and miR-584-5p), and no pathway-level findings can be specifically attributed to miR-155-5p from this study (21) (**Table 4**), (**Figures 3**, **4**).

#### 3.1.4 miR-424-5p in pemphigus: expression profiles, biomarker performance, and mechanistic evidence

##### 3.1.4.1 Expression profiles of miR-424-5p across biological compartments, disease subtypes, and clinical phases

Four studies reporting data on miR-424-5p in pemphigus were included (17, 21, 24, 25).

Wang et al. (17) reported that miR-424-5p exhibited the highest fold change in the entire panel (details previously mentioned) at 1,507.524-fold (*p* = 0.0010). RT-qPCR validation confirmed significantly higher PBMCs miR-424-5p levels in patients compared to HC (p < 0.05).

He et al. (21) reported that miR-424-5p expression level was significantly elevated in PV serum relative to HC (p < 0.001) in the initial screening phase (21).

Valentino et al. (25) reported that miR-424-5p exhibited higher expression in plasma-derived exosomes (P-EVs) from MPV patients compared to HC (p < 0.05) (25). miR-424-5p was the most abundant miRNA species detected among the differentially expressed miRNAs in P-EVs from MPV patients by TaqMan PCR (25). This dysregulated expression pattern was also confirmed in MPV tissue biopsies and in an *in vitro* PV keratinocyte model, showing concordant trends with the plasma exosomal findings (25).

Teplyuk et al. (16) reported a significantly elevated miR-424-5p expression in PBMCs of active disease compared to remission, HC, and other bullous dermatoses (p < 0.0001). No significant differences were observed between the three control groups (p > 0.05) (16). No correlation was reported between miR-424-5p expression and PDAI scores (r = 0.2166, p = 0.198) (16). ROC analysis yielded an AUC of 0.9581, providing a sensitivity of 94.59% and a specificity of 90.00% (16).

##### 3.1.4.2 Treatment response and longitudinal monitoring

Teplyuk et al. (16) assessed miR-424-5p expression before and after three weeks of systemic glucocorticoid therapy in active pemphigus patients. Unlike miR-338-3p, miR-424-5p expression showed no statistically significant change between week 1 and week 3 (8.67 ± 3.58 vs. 9.92 ± 4.92; p = 0.5822), despite a high degree of within-pair data consistency (Spearman rs = 0.7181, p < 0.0001) (16). This finding suggests that, in contrast to miR-338-3p, miR-424-5p expression does not track short-term therapeutic response.

##### 3.1.4.3 Mechanistic evidence: predicted target genes and pathway associations

GO analysis identified 20 significantly enriched biological processes, including intracellular signal transduction, regulation of kinase activity, regulation of protein kinase activity and the MAPK cascade (17). KEGG pathway analysis identified 10 significantly enriched pathways, the most significantly enriched was the Insulin signaling pathway (43 genes, p = 5.4E-8), followed by pathways in cancer, the neurotrophin signaling pathway, the Wnt signaling pathway, and the mTOR signaling pathway, with the T cell receptor signaling pathway (29 genes, p = 3.4E-4) and the MAPK signaling pathway (52 genes, p = 4.8E-3) also among the significantly enriched pathways (17) (**Table 4**). Among the predicted target genes mapped to the MAPK pathway, Wang et al. (17) proposed MAPKAPK3 and CDC25B as candidate regulators of HSP27 phosphorylation downstream of p38 MAPK activation, a process implicated in cytoskeletal reorganization and desmosome disruption in pemphigus keratinocytes (17) (**Figures 3**, **4**).

## 4 Discussion

Beyond confirming miRNA dysregulation in pemphigus, the evidence reviewed here begins to address a clinically unmet need to identify molecular markers that track disease activity, predict treatment response, and illuminate targetable pathogenic pathways.

### 4.1 miR-338-3p

#### 4.1.1 Expression pattern

In this review, miR-338-3p is the most consistently upregulated miRNA across all pemphigus cohorts, with significant elevation in PBMCs confirmed across geographically and methodologically independent Chinese, Russian, and Tunisian datasets (16–20) (24). The magnitude of the fold change, first estimated at ~1,452-fold and the independent replication of strong PDAI, PAAS and ABSIS correlations, collectively suggest that miR-338-3p tracks active immunopathology rather than non-specific inflammation. Notably, the only PF-focused study, Khabou et al. (22), did not detect significant upregulation at baseline, possibly reflecting the distinct Dsg1-centred humoral immunology of PF or limited statistical power in that small cohort. This disease-subtype dependency mirrors observations in other autoimmune diseases: in SLE, circulating miR-338-3p is elevated and mechanistically linked to ERK/MAPK pathway activation (Samir et al., 2025), with similar results reported in myasthenia gravis (27), while in fibroblast-like synoviocytes and terminal ileum epithelial cells from rheumatoid arthritis (RA) and Crohn’s disease (CD) patients, respectively, miR-338-3p is paradoxically downregulated (28, 29). This divergence suggests that the direction of miR-338-3p dysregulation is determined by cell type and local inflammatory context rather than a universal autoimmune signal.

#### 4.1.2 Diagnostic and biomarker potential

The AUC values of 0.8919 and 0.9711 reported in two independent studies position miR-338-3p among the highest-performing single miRNA biomarkers in pemphigus (16, 18). The key clinical implication is not merely its discriminative accuracy against HC, but its earlier therapeutic responsiveness: miR-338-3p declined significantly within two weeks of treatment initiation, preceding any significant change in anti-Dsg3 antibody titers (18). This kinetic advantage makes it a potentially superior pharmacodynamic readout compared to current serological standards. Furthermore, the differential response between glucocorticoid responders and non-responders documented by Xu et al. (20) introduces a prognostic dimension; persistent elevation after treatment could flag inadequate immunological response earlier than conventional monitoring. However, the claim of its potential as a diagnostic biomarker requires an important caveat; He et al. (21) found that serum miR-338-3p was not discriminating against MMP (21), suggesting its elevation may not be pemphigus-specific within the broader blistering disease spectrum. A transcriptome analysis in type 1 diabetes (T1D) (30) similarly framed miR-338-3p as a ‘progressive biomarker’ rather than a disease-specific signal, reinforcing the need for active disease as a comparator group beyond HCs in future diagnostic studies.

#### 4.1.3 Mechanisms and target genes

Three direct molecular targets have each been independently validated, RNF114 (18), TRADD (19), and RUNX1, collectively sketching a model in which elevated miR-338-3p simultaneously disrupts T-cell activation through RNF114 (previously identified as a positive regulator of T cell activation, with its overexpression promoting T cell activation in a dose-dependent manner (31)), skews cytokine balance toward a Th2/pro-autoantibody environment through TRADD suppression, leading to decreased IFN-γ and elevated IL-4 and IL-10, and impairs Treg-mediated peripheral tolerance through downregulation of RUNX1, which in turn reduces FOXP3 expression. Together, these axes provide a mechanistic basis for how a single miRNA could coordinate autoreactive B-cell expansion and anti-Dsg antibody production in PV. A critical limitation, however, is that the mechanistic model, while internally coherent, awaits independent replication. Additionally, the paradoxical *in vivo* upregulation of RNF114 protein despite elevated miR-338-3p in PV patients could be attributed to a compensatory feedback mechanism as suggested by the authors, but also highlights the gap between *in vitro* transfection systems and native *in vivo* regulatory contexts, a challenge well-recognized in miRNA biology that underscores the necessity of *in vivo* validation before mechanistic conclusions can inform therapeutic design.

### 4.2 miR-146a-5p

#### 4.2.1 Expression pattern

In pemphigus, miR-146a-5p is consistently upregulated in lesional skin of chronic moderate-to-severe PF patients but not reliably in peripheral blood. This compartment-specificity is biologically meaningful: miR-146a functions as a negative feedback regulator of TLR-driven NF-κB signaling via the TRAF6/NF-κB axis (32, 33), suggesting that its tissue-level elevation reflects a compensatory response to local inflammation rather than primary pathogenesis. The broader autoimmune literature supports this context-dependency, with miR-146a-5p showing divergent expression across SLE, lupus nephritis (LN), Sjogren syndrome (SS), T1D and multiple sclerosis (MS) depending on tissue compartment and disease phase (32, 34–37). Consequently, blood-based assays are unlikely to reliably capture its dysregulation in pemphigus.

#### 4.2.2 Diagnostic and biomarker potential

No ROC analysis was performed for miR-146a-5p in either included pemphigus study, and its cutaneous expression showed no significant correlation with PDAI scores, precluding any formal assessment of diagnostic utility. The gap is particularly notable given the substantial diagnostic performance documented across other autoimmune and inflammatory conditions: AUCs of 0.857 in SLE (37), 0.80 in RA (38), and exceeding 0.99 in psoriasis; itself a chronic inflammatory skin disease; where miR-146a-5p effectively tracked adalimumab response (39). Importantly, even within SLE the discriminatory ability varies considerably by cohort, with Sadaty et al. (2026) reporting an AUC of only 0.299 (40), underscoring that diagnostic performance is context- and compartment-dependent. Formal validation in pemphigus, incorporating predefined ROC analysis, tissue-versus-blood comparisons, and disease-matched controls, is therefore required before miR-146a-5p can be meaningfully considered for clinical translation.

#### 4.2.3 Mechanisms and target genes

The mechanistic evidence for miR-146a-5p in pemphigus rests entirely on in silico pathway enrichment, with TRAF6 and IRAK1 identified as the principal predicted targets within the Toll receptor signaling pathway. While unvalidated in pemphigus-specific cells, these predictions are biologically credible: the suppressive axis whereby miR-146a-5p downregulates TRAF6 and IRAK1, thereby attenuating NF-κB activation, is among the most consistently replicated mechanisms in autoimmune biology, confirmed across keratinocytes, HUVECs, and intestinal epithelial cells, and operative across RA, SLE, inflammatory bowel disease (IBD), and psoriasis (41–46). The authors proposed that miR-146a-5p–mediated suppression of PTGS2 may contribute to reduced inflammation in chronically treated patients (22); since PTGS2 (COX-2) is itself an NF-κB-responsive gene, this observation is consistent with, rather than separate from, the TRAF6/IRAK1 suppressive axis described above. The predicted antagonism between miR-146a-5p and miR-155-5p through shared TRAF6/MyD88 targets is consistent with their established counter-regulatory roles in innate immunity. While miR-155-5p promotes inflammatory responses by facilitating the TLR4/MyD88/NF-κB pathway (47, 48), miR-146a-5p provides a critical brake on this system (49). This interplay has emerged as particularly significant in multiple disease contexts, from ulcerative colitis (49) to bacterial infections (50) and even cancer-related inflammation (51), highlighting the importance of this balanced regulatory network in maintaining immune homeostasis. These mechanistic models remain inferential and require direct experimental validation in pemphigus.

### 4.3 miR-155-5p

#### 4.3.1 Expression pattern

miR-155-5p is consistently upregulated in active pemphigus across both serum and lesional skin, with cutaneous expression tracking disease phase: highest in active and chronic disease, lowest in clinical remission. The absence of significant dysregulation in blood despite marked tissue-level elevation mirrors the compartment-sensitivity pattern observed for miR-146a-5p, reinforcing that blood-based assays systematically underestimate miRNA dysregulation occurring at the lesional level. An early signal from a distinct cellular compartment was reported by Cipolla and Luiza (2013) in a conference abstract where miR-155 was 2-fold upregulated in IgG^+^ B cells of inactive PF patients relative to HC (p = 0.009), with a non-significant trend toward higher expression in inactive versus active disease (p = 0.082) (26). Although these findings derive from a conference abstract and therefore carry limited evidentiary weight, the cell-type specificity of this signal, confined to IgG+ B cells rather than whole blood or tissue, introduces a lymphocyte-compartment dimension absent from the two included peer-reviewed studies and warrants formal follow-up. This upregulation pattern aligns with the broader autoimmune literature, where miR-155-5p is aberrantly expressed across RA (52), MS (53) and systemic sclerosis (SSc) (54), and T1D (55, 56), suggesting a contributory role of miR-155-5p in the pathogenesis of AIDs.

#### 4.3.2 Diagnostic and biomarker potential

The diagnostic performance of serum miR-155-5p in distinguishing PV from MMP (21) is clinically significant because it reflects discrimination between two clinically overlapping blistering diseases, a considerably relevant benchmark. This distinction is particularly important because PV and MMP can present with similar mucocutaneous blistering, yet differ markedly in their underlying pathophysiology, prognosis, and therapeutic management. Accurate differentiation is therefore essential to guide appropriate treatment strategies and avoid mismanagement, especially in early or atypical presentations. Furthermore, the serial combination with miR-584-5p, achieving 100% specificity, further supports the principle that miRNA panels outperform individual markers in diagnostically ambiguous cases. At the tissue level, the correlation between cutaneous miR-155-5p and PDAI positions it as a disease activity monitor rather than merely a diagnostic marker, a dual utility that few candidate biomarkers in pemphigus currently demonstrate. A structural parallel is seen in alopecia areata (AUC = 0.97 vs. SALT scores) (57), reinforcing that miR-155-5p achieves reproducibly high diagnostic metrics across autoimmune skin diseases. Prospective validation incorporating multicompartment sampling, serum, skin, and blister fluid, with standardized cutoff values and direct comparison against established serological markers such as anti-Dsg Abs titers remain necessary before clinical translation.

#### 4.3.3 Mechanisms and target genes

The pathway enrichment profile of miR-155-5p, spanning the PI3K and p53 feedback loop 2, and Hedgehog signaling pathways. PI3K pathway enrichment is particularly compelling given that its negative regulators, SHIP-1 and PTEN, are established miR-155 targets in RA (58) and psoriasis (59) respectively, where their suppression sustains pathogenic lymphocyte survival and inflammatory signaling. The enrichment of the p53 feedback loop 2 pathway suggests that miR-155-5p may contribute to pemphigus pathogenesis through modulation of p53-dependent regulatory networks, potentially contributing to both the persistence of autoreactive immune responses and the keratinocyte apoptotic cascade central to pemphigus pathology. In pemphigus, p53 is a well-established mediator of keratinocyte stress responses, participating in a self-amplifying pathomechanism whereby *DSG3* loss activates p53 via p38MAPK, which in turn drives ST18 overexpression and further perpetuates acantholysis and autoantibody-mediated DSG3 downregulation (60–62). All mechanistic claims in pemphigus remain inferential and require direct experimental validation.

### 4.4 miR-424-5p

#### 4.4.1 Expression pattern

miR‑424‑5p was consistently upregulated in PV and MMP across multiple compartments, including PBMCs (16, 17), serum (21), and plasma‑derived exosomes (25), confirming its robust elevation in these autoimmune blistering diseases. This pattern is not unique to pemphigus; similar upregulation of miR‑424‑5p in PBMCs has been reported in ankylosing spondylitis (63), RA (64), and T1D (65), suggesting that its increase in circulating immune cells represents a conserved signature of systemic immune activation. However, the expression of miR‑424‑5p is not universally elevated across all tissues or disease contexts. In SS, for instance, it is actively suppressed in salivary gland epithelium under chronic IFN‑γ stimulation (66), highlighting a striking compartment‑specific divergence. Collectively, these findings indicate that, while miR‑424‑5p in peripheral blood components may serve as a sensitive biomarker of immune activation in pemphigus and other inflammatory conditions, its regulatory direction is highly dependent on the interplay between cell type, local microenvironment, and cytokine milieu. Therefore, the interpretation of miR‑424‑5p as a biomarker must consider both the compartment analyzed and the underlying disease context.

#### 4.4.2 Diagnostic and biomarker potential

In pemphigus, Teplyuk et al. (16) established an AUC of 0.93 for miR-424-5p with a significant positive correlation with PDAI scores (16), indicating that its elevation reflects not only disease presence but also disease burden, a clinically meaningful property for activity monitoring. The study’s inclusion of other bullous dermatoses as comparators, rather than HC alone, strengthens the specificity of this estimate, though its preprint status warrants caution. Beyond pemphigus, miR-424-5p demonstrates consistently strong diagnostic performance across autoimmune diseases: an AUC of 0.966 with 90.9% sensitivity and 87.5% specificity in T1D (65), and an AUC of 0.94 in alopecia areata (67). This cross-disease discriminative capacity, while attesting to its biological relevance in systemic autoimmunity, simultaneously limits its disease specificity as a standalone pemphigus biomarker.

#### 4.4.3 Mechanisms and target genes

Bioinformatic analyses by Wang et al. (17) proposed the implication of miR-424-5p in T-cell receptor and MAPK signaling (17). This mechanistic hypothesis is reinforced by independent cross-disease evidence: in acquired aplastic anemia, miR-424-5p functions as a core node in a bone marrow T-cell miRNA-mRNA network, with KEGG analysis linking its dysregulated targets to MAPK signaling and T-cell differentiation (68). Beyond MAPK, miR-424-5p has been shown to regulate PD-L1 expression in T cells, thereby promoting IFN-γ and TNF-α secretion. In psoriasis, miR-424-5p participates in the AGAP2-AS1/miR-424-5p/SGK1 axis, through which exosomal lncRNA drives Th1 and Th17 differentiation in CD4^+^ T cells via SGK1, a downstream effector of T-cell receptor signaling (69), a mechanism conceptually relevant to the CD4^+^ T-cell dysregulation documented in pemphigus. Additionally, in RA synovial fibroblasts, elevated miR-424 engages a DICER1-dependent feedback loop that perpetuates broader miRNA dysregulation (64), raising the possibility that analogous amplification mechanisms operate in pemphigus. Critically, none of these target interactions have been experimentally validated in pemphigus cells, representing the most significant mechanistic gap and a clear priority for future functional studies.

### 4.5 Other potentially relevant miRNAs in pemphigus

Although the present review focused on miR-338-3p, miR-146a-5p, miR-155-5p and miR-424-5p on the basis of our pre-defined eligibility criteria, several additional miRNAs may also be relevant to pemphigus pathogenesis and biomarker development. miR-125b-5p, miR-181a-5p, miR-181b-5p, miR-326 and miR-584-5p have been reported as upregulated in PV, and their combined target network implicates T-cell receptor, p53, p38-MAPK and cell-adhesion signaling pathways (21). miR-584-5p may additionally regulate KLRG1, with disruption of its binding site associated with PF susceptibility (70). In PF, miR-21-5p and miR-17-5p also show altered expression (22). miR-148a-3p is downregulated in MPV and has been functionally linked to MMP7 and keratinocyte adhesion (25). More recently, miR-125b-5p has been reported to regulate the p63/PERP axis, providing a potential mechanism linking miRNA dysregulation to desmosomal impairment (71). These findings further support the investigation of these miRNAs as additional components of the regulatory network underlying pemphigus.

### 4.6 Future perspectives and limitations

Despite the growing body of evidence supporting the involvement of miRNAs in pemphigus pathogenesis, the current literature remains fragmented and limited by several methodological and conceptual constraints. Most studies are based on relatively small cohorts, which restricts statistical power and generalizability across ethnic groups and pemphigus subtypes. Considering the well-established genetic heterogeneity of pemphigus, particularly in endemic variants, large multicenter and multiethnic studies are required to validate the diagnostic and prognostic value of candidate miRNAs such as miR-338-3p, miR-146a-5p, miR-155-5p, and miR-424-5p.

Another important limitation is the heterogeneity of biological samples analyzed across studies (serum, PBMCs, isolated lymphocyte subsets, lesional skin, exosomes). While this diversity provides complementary insights, it also complicates direct comparisons and may explain contradictory findings. Standardization of sample processing, normalization strategies, and quantification methods is essential before clinical translation can be considered.

Mechanistically, most proposed miRNA–target interactions rely heavily on bioinformatic predictions or indirect associations. Although some targets such as RUNX1 (miR-338-3p), TRAF6 (miR-146a), and SOCS1 (miR-155) have been experimentally validated in other autoimmune contexts, robust functional validation in pemphigus-specific cellular systems remains scarce. Future studies should incorporate *in vitro* keratinocyte–immune cell co-culture models, CRISPR/Cas-based gene modulation, and humanized mouse models to dissect causal relationships rather than correlations.

In addition, future mechanistic studies should investigate whether dysregulated miRNAs modulate B-cell survival and differentiation pathways, including BAFF and APRIL signaling, which are known to support autoreactive B-cell maintenance in pemphigus.

Importantly, the temporal dynamics of miRNA expression during different disease phases, preclinical autoimmunity, flare, remission, and relapse, remain poorly characterized. Longitudinal studies integrating miRNA profiling with validated clinical scoring systems (e.g., PDAI and ABSIS), autoantibody titers, and therapeutic response (particularly to corticosteroids and biologics such as rituximab) could clarify whether specific miRNAs serve as predictive biomarkers rather than merely activity markers.

From a translational perspective, miRNA-based therapeutics represent an exciting but still theoretical avenue in pemphigus. Strategies such as antagomiRs, miRNA mimics, or exosome-mediated delivery systems may allow targeted modulation of pathogenic pathways, including Treg dysfunction and Th1/Th2/Th17 imbalance.

## 5 Conclusion

Accumulating evidence indicates that microRNAs are integral regulators of immune dysregulation in pemphigus. Among the miRNAs reviewed, miR-338-3p emerges as the most consistently validated biomarker, demonstrating strong associations with disease activity, autoantibody production, Treg dysfunction, and therapeutic response. miR-146a-5p and miR-155-5p appear to participate in finely tuned inflammatory feedback networks, particularly involving NF-κB, TRAF6, IRAK1, and SOCS1 pathways, whereas miR-424-5p may contribute to pathogenic signaling cascades linked to T-cell receptor and MAPK activation. Together, these miRNAs form interconnected regulatory circuits influencing immune tolerance, inflammatory amplification, and keratinocyte adhesion integrity.

Beyond their diagnostic utility, these miRNAs provide mechanistic insights into the breakdown of immune tolerance that characterizes pemphigus. Their involvement in Treg impairment, Th cell polarization, cytokine signaling, and inflammasome regulation underscores the multifactorial nature of disease pathogenesis, bridging genetic susceptibility and environmental triggers through epigenetic modulation.

In conclusion, while the current evidence remains preliminary, the integration of miRNA biology into pemphigus research represents a critical step toward precision medicine in autoimmune blistering diseases. Continued mechanistic and translational investigations will determine whether modulation of specific miRNA networks can evolve from a biomarker strategy into a viable therapeutic paradigm.

## Data Availability

The original contributions presented in the study are included in the article/supplementary material. Further inquiries can be directed to the corresponding author/s.
